# First submicroscopic inversion of the *OPA1* gene identified in dominant optic atrophy – a case report

**DOI:** 10.1186/s12881-020-01166-z

**Published:** 2020-11-26

**Authors:** Nicole Weisschuh, Pascale Mazzola, Tilman Heinrich, Tobias Haack, Bernd Wissinger, Felix Tonagel, Carina Kelbsch

**Affiliations:** 1 Institute for Ophthalmic Research, Centre for Ophthalmology, University of Tübingen, Tübingen, Germany; 2 Institute of Medical Genetics and Applied Genomics, University of Tübingen, Tübingen, Germany; 3 Centre for Rare Diseases, University of Tübingen, Tübingen, Germany; 4 University Eye Hospital, Centre for Ophthalmology, University of Tübingen, Tübingen, Germany

**Keywords:** Case report, Inversion, Complex rearrangement, Dominant optic atrophy, *OPA1*, Non-homologous end joining (NHEJ)

## Abstract

**Background:**

Dominant optic atrophy (DOA) is an inherited optic neuropathy that mainly affects visual acuity, central visual fields and color vision due to a progressive loss of retinal ganglion cells and their axons that form the optic nerve. Approximately 45–90% of affected individuals with DOA harbor pathogenic variants in the *OPA1* gene. The mutation spectrum of *OPA1* comprises nonsense, canonical and non-canonical splice site, frameshift and missense as well as copy number variants, but intragenic inversions have not been reported so far.

**Case presentation:**

We report a 33-year-old male with characteristic clinical features of DOA. Whole-genome sequencing identified a structural variant of 2.4 kb comprising an inversion of 937 bp at the *OPA1* locus. Fine mapping of the breakpoints to single nucleotide level revealed that the structural variation was an inversion flanked by two deletions. As this rearrangement inverts the entire first exon of *OPA1*, it was classified as likely pathogenic.

**Conclusions:**

We report the first DOA case harboring an inversion in the *OPA1* gene. Our study demonstrates that copy-neutral genomic rearrangements have to be considered as a possible cause of disease in DOA cases.

**Supplementary Information:**

The online version contains supplementary material available at 10.1186/s12881-020-01166-z.

## Background

Dominant optic atrophy (DOA, MIM#165500) and Leberʼs hereditary optic neuropathy (LHON, MIM#535000) are the two most common entities of inherited optic neuropathies seen in clinical practice [1]. DOA was first described in the 1950s and is genetically and clinically distinct from LHON [2, 3]. Clinically, affected individuals with DOA present with temporally accented pallor of the optic nerve head at fundus examination, and bilateral central or caeco-central scotoma at visual field examination. A laterally symmetrical temporal reduced thickness of the retinal nerve fiber layer (RNFL) is seen upon optical coherence tomography imaging (OCT). Other than in affected individuals diagnosed with LHON, who often show red-green dyschromatopsia, the color vision defect in DOA reflects a generalized dyschromatopsia or is specific to the tritan axis [4]. Visual acuity can range from 20/20 to light perception, with 40% of affected individuals having a visual acuity over 20/60 [5].

Depending on the population studied, 45–90% of DOA cases harbor pathogenic variants in *OPA1* [6, 7], which was the first gene to be described as an underlying cause of DOA [8, 9]. *OPA1* encodes a dynamin-related GTPase which is imported into mitochondria and plays an important role in mitochondrial dynamics and structural maintenance of the cristae junctions [10, 11]. As of August 2020, the Human Gene Mutation Database (HGMD) lists 404 disease-causing variants in *OPA1*. Most variants are private and only few constitute founder alleles [7, 12]. Overall, the majority of disease-causing variants are loss-of-function alleles, indicating that haploinsufficiency is the predominant disease mechanism underlying *OPA1*-linked DOA [12, 13]. The mutation spectrum comprises nonsense, canonical splice site, frameshift and missense variants. In addition, copy number variants (CNVs) and deep-intronic variants inducing aberrant splicing have been reported [14–17], necessitating a comprehensive genetic diagnostic testing including the analysis of read depth and intronic sequences. So far, submicroscopic inversions in the *OPA1* gene have not yet been reported as a cause of DOA. Since inversions are copy-neutral, they escape detection by conventional diagnostic technologies such as microarrays and read depth methods. We here present the first affected individual with DOA in whom whole genome sequencing based on short read technology led to the identification of a complex structural rearrangement including the first coding exon of *OPA1*. Our findings demonstrate that copy-neutral genomic rearrangements have to be considered as a possible cause of disease in DOA.

## Case presentation

The 33-year-old male subject of German descent presented with a history of bilateral slowly progressive vision loss. His younger brother was reported to have “bad vision” but was not available for phenotypic and genetic analysis. Parents and grandparents were apparently unaffected. Cerebral imaging (magnetic resonance imaging with contrast) had not revealed any underlying cause, particularly no space-occupying lesions compromising the visual pathway.

### Clinical investigation

At examination, best-corrected visual acuity was 0.5 (right eye) and 0.4 (left eye). According to previous eye examinations, visual acuity was documented to have been 0.8 in both eyes 10 years ago with a continuous slowly progressive decline.

**Fig. 1:**
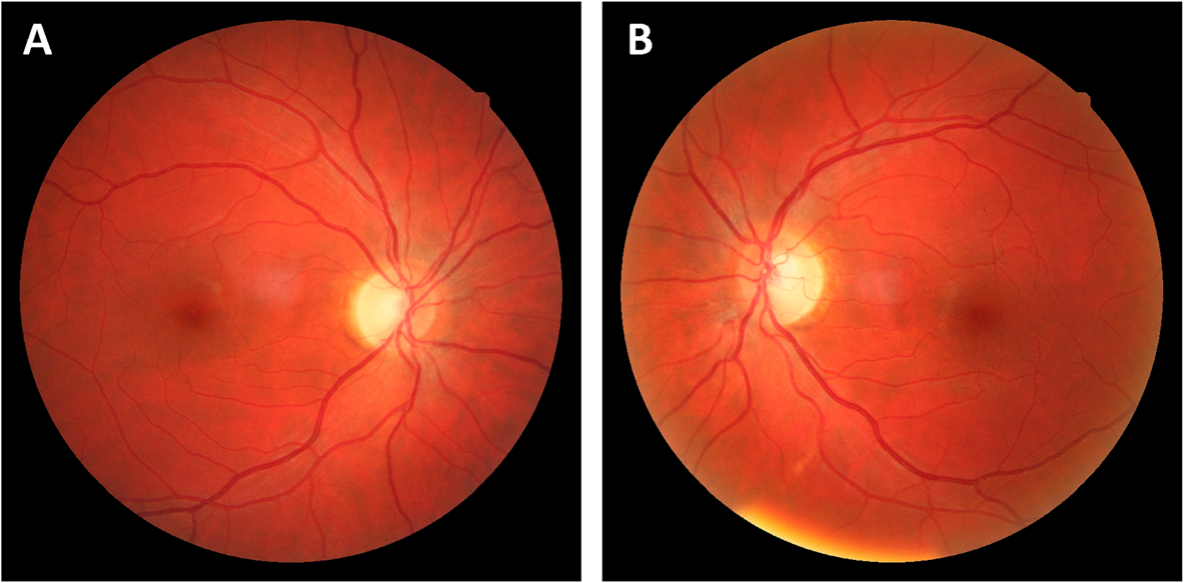
Eye fundi. Temporal pallor of discs is seen in the right eye (**a**) and the left eye (**b**) of the affected proband

**Fig. 2:**
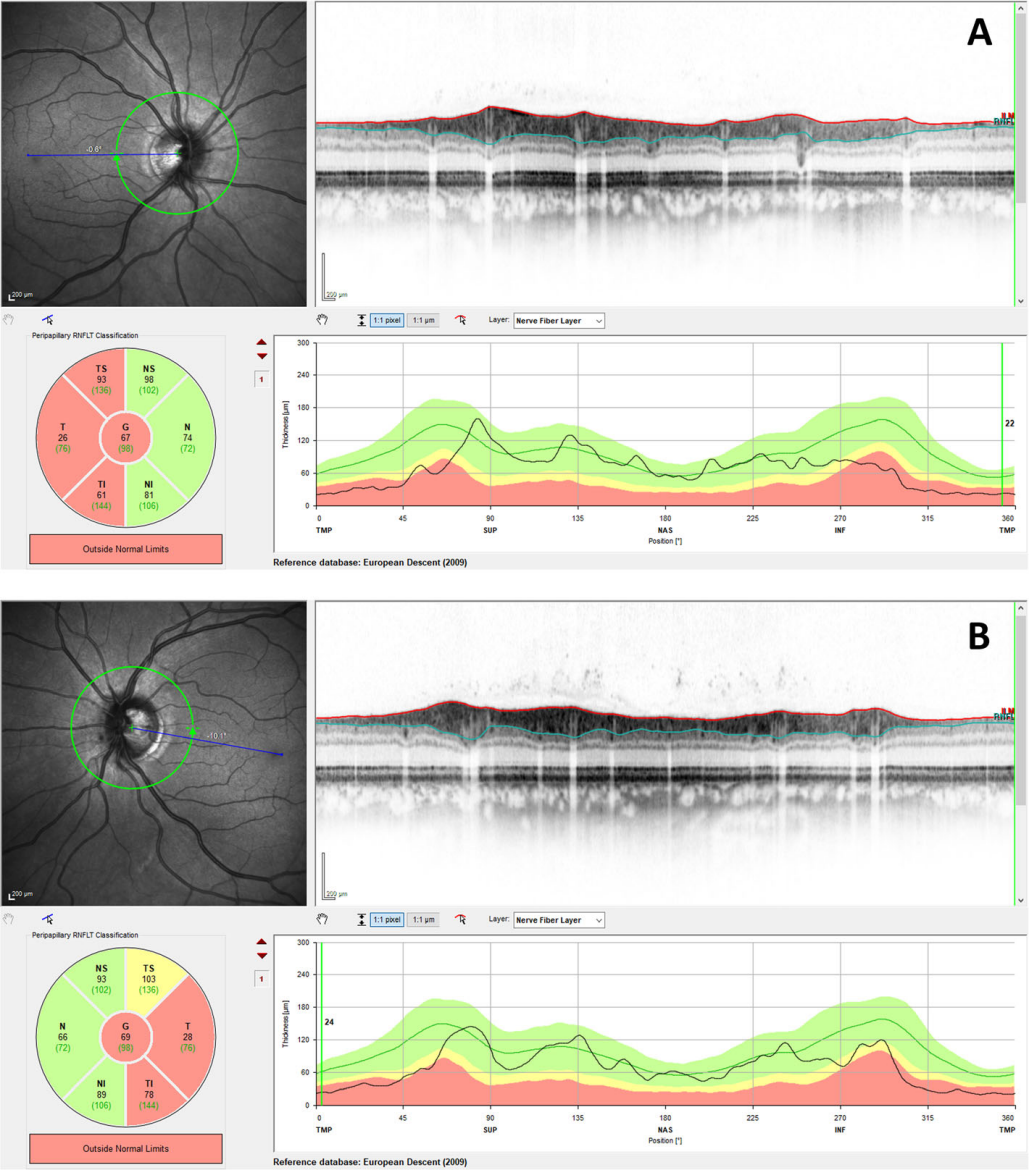
Optical coherence tomography (OCT) of the retinal nerve fiber layers (RNFLs). The right eye (**a**) and left eye (**b**) OCT of the affected proband show temporal thinning of RNFLs

Anterior segment slit-lamp examination was unremarkable. Intraocular eye pressure was within normal limits, and no relative afferent pupillary defect was found. Fundus ophthalmoscopy unveiled bilateral symmetric, temporally accented optic disc pallor, which is a typical and characteristic finding of DOA (Fig. 1). Correspondingly, the temporal optic atrophy could be confirmed by bilaterally reduced RNFL in OCT (Fig. 2).

Visual field examination of the central 30° of the visual field was relatively unremarkable with only slight paracentral relative defects R > L (Additional file 1). Color testing with Panel-D15 was significant for a dyschromatopsia to the tritan axis L > R (Additional file 2).

In summary, the affected individual presented with characteristic clinical findings consistent with a diagnosis of DOA.

### Genetic analysis

**Fig. 3:**
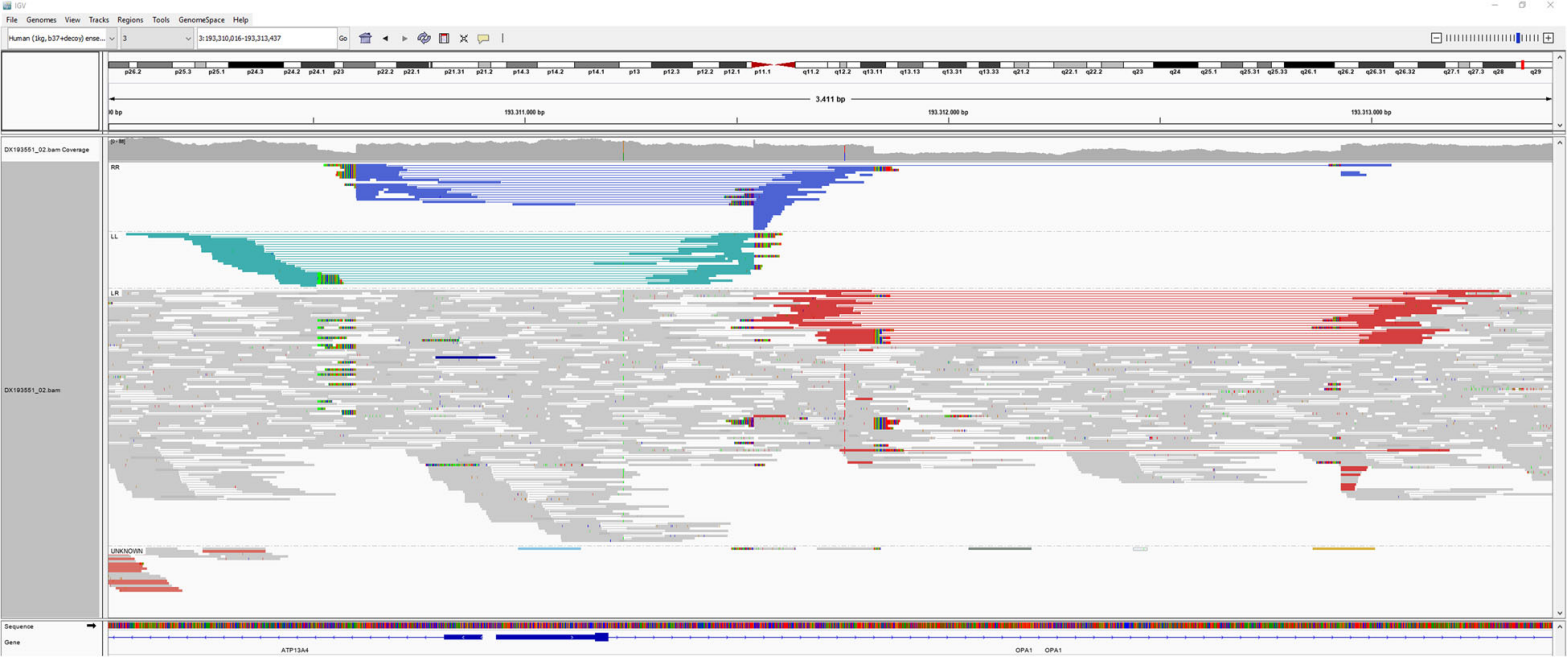
Integrative Genomics Viewer (IGV) screenshot of the genomic region containing the structural variant identified in the affected proband. From top to bottom the read coverage track, the alignment track (reads colored by insert size and pair orientation and grouped by pair orientation) and the gene track are shown. Colored reads represent discordant reads with unexpected insert size and/or pair orientation when aligned to the reference genome indicating the structural variant. Blue read pairs/lines represent read pairs with right-right pair orientation, teal reads represent read pairs with left-left pair orientation and red read pairs/lines represent read pairs with aberrant insert size. Split reads with soft-clipped bases span the breakpoints of the structural variant

**Fig. 4:**
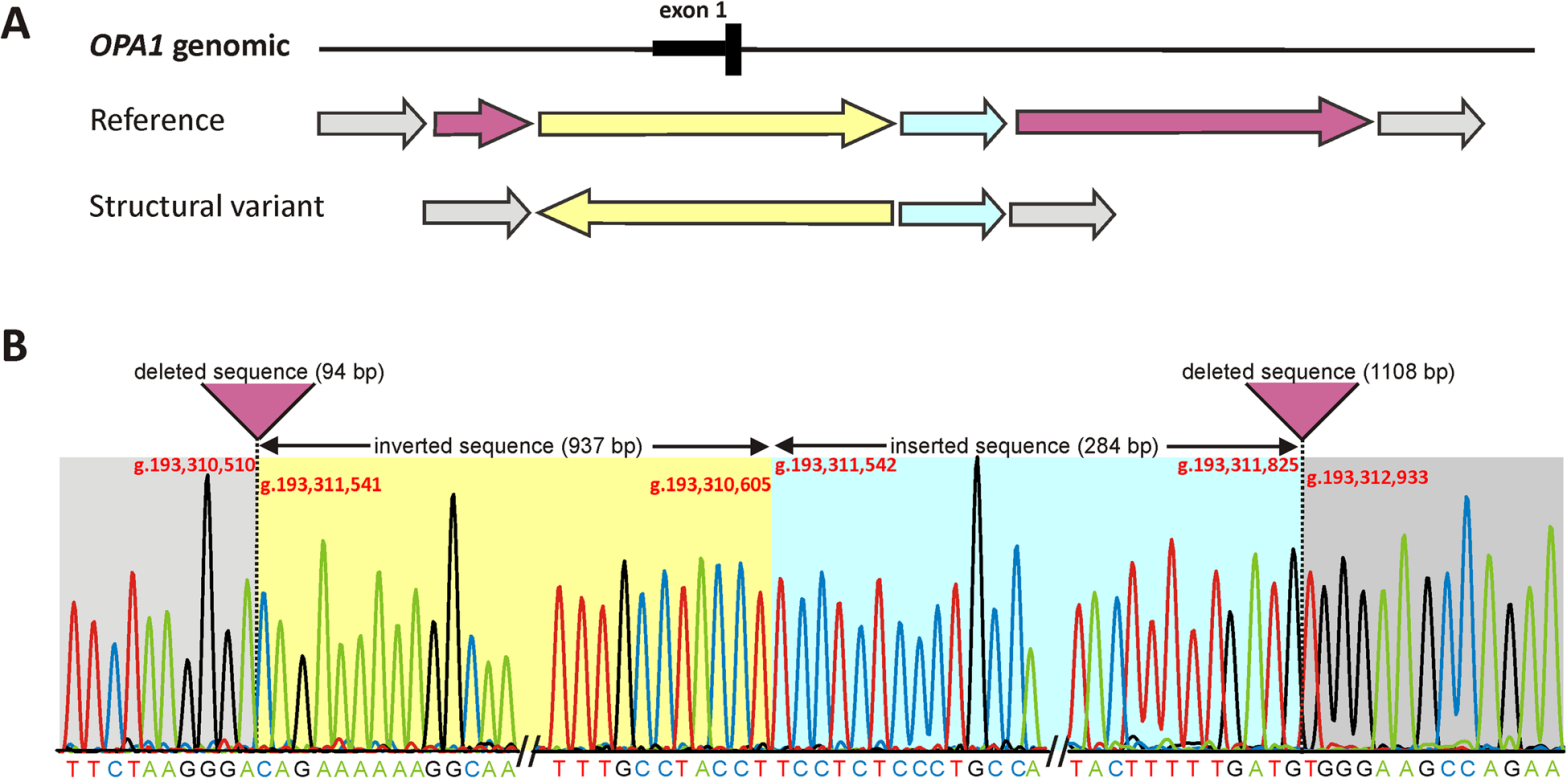
Fine mapping of the structural variant by sequencing of breakpoint junctions. **a** Schematic representation of the structural variant. The color of the arrows indicates normal sequence (grey), inverted sequence (yellow), inserted sequence (blue) and deleted sequence (pink). **b** Sanger sequencing of breakpoint amplicons. The vertical dashed lines highlight the 5´ and 3′ breakpoints. The background colors of the electropherogram correspond to the scheme shown in (A). The deleted sequences are indicated by pink triangles. Note that the inserted sequence is actually “leftover original sequence” between the deletion and inversion events

Genomic DNA was extracted from peripheral blood using standard protocols. Whole genome sequencing (2 × 150 bp paired-end reads) was performed on an Illumina platform (NovaSeq6000). The average coverage on target was 54.8x (99.76% > 20x). Bioinformatic processing of raw read data, annotation and variant calling was performed as described previously [18]. For details refer to the megSAP pipeline (https://github.com/imgag/megSAP) developed at the Institute of Medical Genetics and Applied Genomics, University Hospital Tübingen, Germany. Bioinformatic analysis and genomic coordinates given in this manuscript are based on the GRCh37 genome (hg19). The Manta Structural Variant Caller [19] identified a ~ 1 kB spanning inversion that changes the orientation of the first coding exon of *OPA1*. Manual inspection of split reads using the Integrative Genomics Viewer (IGV, version 2.3, see Fig. 3) revealed a complex rearrangement. Subsequent breakpoint PCR and Sanger sequencing was performed in order to resolve the variant configuration to single nucleotide resolution. Figure 4a gives a schematic overview of the structural variant, and Fig. 4b shows the Sanger sequencing of the breakpoint junctions. The nomenclature for the variant according to the guidelines established by the Human Genome Variation Society (HGVS) [20] was established as follows: NC_000003.11:g.193,310,511_193,312,932delins193,310,605_193,311,825[193,310,605_193,311,541inv].

It was not possible to obtain an RNA sample from the proband, excluding transcript analysis. However, since the inversion comprises the first coding exon of the *OPA1* gene including the translational start site, it most likely constitutes a loss-of-function allele.

## Discussion and conclusions

This is the first report of a submicroscopic inversion involving the *OPA1* gene. Inversions are mainly mediated by three mechanisms: While non-allelic homologous recombination (NAHR) and the fork stalling and template switching (FoSTeS) mechanism require homologous sequences at the breakpoints, non-homologous end joining (NHEJ) is a repair mechanism for double-strand breaks [21–24]. In the latter, the inverted sequence directly ligates to the breakpoint, independent of sequence homology. Using the online tools Blast 2 Sequences (http://polyp.biochem.uci.edu/blast/wblast2.html) and Repeat Masker (http://www.repeatmasker.org), we analyzed the sequences flanking the breakpoints for similarities to each other and to repeat elements that can mediate genomic rearrangements (data not shown). The lack of sequence homology at the breakpoints and the sequencing results of breakpoint junctions indicate that the structural variant described in this study most likely involved multiple double-strand breaks and the rejoining of DNA fragments by NHEJ. Of note, we followed the guidelines of the HGVS when assigning the nomenclature to the variant. However, from a mechanistic point of view, the inserted sequence is basically “leftover original sequence” between the deletion and inversion events.

Screening for the structural variant in a cohort of 350 optic atrophy cases that had been previously tested negative for single nucleotide variants and CNVs in *OPA1* was performed using patient-specific breakpoint PCRs (primer pairs OPA1-Inv-BP1-f: 5′-tgctcagcactaggcatctg-3′ / OPA1-Inv-BP1-r: 5′-ttctggacgcctctcaatct-3′, OPA1-Inv-BP2-f: 5′-ggaacgggaagggctaaa-3′ / OPA1-Inv-BP2-r: 5′-tcgaatcaccgtctctgaca-3′, and OPA1-Inv-BP3-f: 5′-tgtcttctttttcttccatttccac-3′ / OPA1-Inv-BP3-r: 5′-atgcttatgctctcatctgttaggg-3′, respectively). All patient-specific breakpoint PCRs were performed as a duplex PCR reaction with primers that bind to the reference sequence and amplify exon 1 of *OPA1* (OPA1-Ex1-F: 5′-actgagtacgggtgcctgtc-3′ / OPA1-Ex1-R: 5’gccagattagagcctgcactt-3′). PCR products were resolved on a 2% agarose gel. No additional cases could be identified among the 350 unsolved cases. The variant was also absent in our internal cohort of 1400 genomes, and in the public databases gnomAD, ClinVar and DECIPHER. The lack of additional cases indicates that the variant is private, and is in line with the proposed molecular mechanism, since recurrent genomic rearrangements are most often mediated by NAHR, and not by NHEJ [25]. DNA samples were not available for familial co-segregation analysis. Hence, we could not establish whether the rearrangement was a de novo event in our proband.

Compared to single nucleotide variants and small insertions and deletions, the mapping of structural variants (SVs) is much more challenging as they cover a large portion of a read or are even larger than the read length [26]. As a consequence, the importance of SVs in Mendelian diseases is not well defined. In a recent comprehensive study, an average of 27,622 SVs was identified per human genome, including 156 inversions per genome, many of which intersected with genomic regions associated with genetic disease syndromes [27]. Whole genome sequencing detects SVs more reliably than exon-based approaches since most of the functionally relevant junctions will have both ends contained within introns or intergenic regions. Given that sequencing costs are constantly decreasing, whole genome sequencing is commissioned into routine clinical care pathways more and more frequently, offering the potential to detect SVs. Third-generation sequencing technologies will probably further improve the diagnostic sensitivity as long read lengths are more likely to contain the whole SV (i.e. both breakpoints), providing less potential for error and facilitating mapping. Future studies will show whether the feasibility to detect copy-number neutral rearrangements can boost the diagnostic rate in Mendelian disorders like DOA.

## Supplementary Information

**Additional file 1:** **Figure 1.** Results of perimetry.

**Additional file 2:** **Figure 2.** Results of panel D15 test.

## Data Availability

The data that support the findings of this study are available from the Institute for Medical Genetics and Genomics (IMGAG) in Tübingen but restrictions apply to the availability of these data, which were used under license for the current study, and so are not publicly available. Data are however available from the authors upon reasonable request and with permission of the IMGAG. The datasets analysed during the current study are available at gnomAD https://gnomad.broadinstitute.org/, ClinVar (https://www.ncbi.nlm.nih.gov/), and DECIPHER (https://decipher.sanger.ac.uk/). The accession numbers used in this study and obtained from the National Center for Biotechnology Information (NCBI) include: NM_015560.2, and the NCBI37/hg19 assembly (https://www.ncbi.nlm.nih.gov/assembly/GCF_000001405.13/).

## Abbreviations

CNV: Copy number variant
DOA: Dominant optic atrophy
FoSTeS: Fork stalling and template switching
IGV: Integrative Genomics Viewer
LHON: Leber’s hereditary optic neuropathy
NAHR: Non-allelic homologous recombination
NHEJ: Non-homologous end joining
OCT: Optical coherence tomography imaging
RNFL: Retinal nerve fiber layer
SV: Structural variant
