# Supplementary figures and images for: First submicroscopic inversion of the OPA1 gene identified in dominant optic atrophy – a case report
Source: BMC Med Genet. 2020 Nov 26;21:236. doi: 10.1186/s12881-020-01166-z (PMC7690134; doi:10.1186/s12881-020-01166-z)

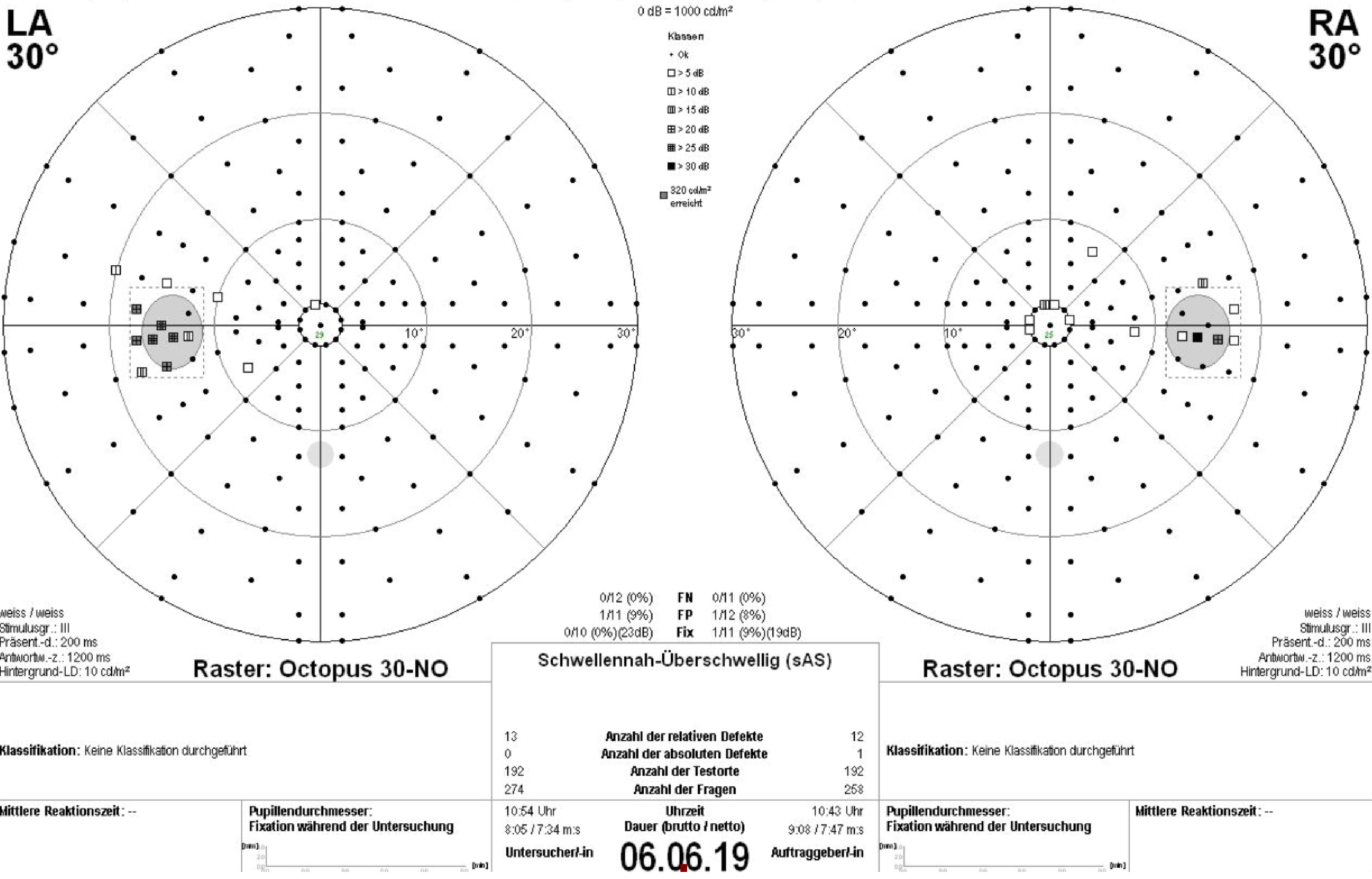

Supplement: Supplementary file 1 — Additional file 1: Figure 1. Results of perimetry. [file 12881_2020_1166_MOESM1_ESM.tif]

1. Lanthony Panel D-15 [desaturiert]

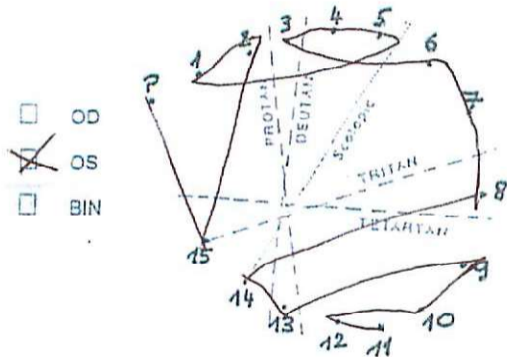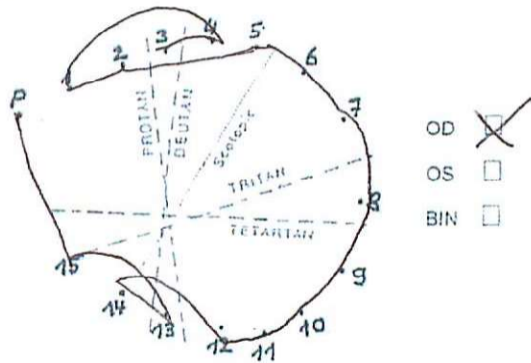

Supplement: Supplementary file 2 — Additional file 2: Figure 2. Results of panel D15 test. [file 12881_2020_1166_MOESM2_ESM.pdf]
